# Overexpression of Two CCCH-type Zinc-Finger Protein Genes Leads to Pollen Abortion in *Brassica campestris* ssp. *chinensis*

**DOI:** 10.3390/genes11111287

**Published:** 2020-10-29

**Authors:** Liai Xu, Tingting Liu, Xingpeng Xiong, Weimiao Liu, Youjian Yu, Jiashu Cao

**Affiliations:** 1 Laboratory of Cell & Molecular Biology, Institute of Vegetable Science, Zhejiang University, Hangzhou 310058, China; 11416052@zju.edu.cn (L.X.); 11416009@zju.edu.cn (T.L.); xiongxingpeng1989@163.com (X.X.); 11616044@zju.edu.cn (W.L.); 2 Key Laboratory of Horticultural Plant Growth, Development and Quality Improvement, Ministry of Agriculture/Zhejiang Provincial Key Laboratory of Horticultural Plant Integrative Biology, Hangzhou 310058, China; 3 Department of Horticulture, College of Agriculture and Food Science, Zhejiang A & F University, Lin’an 311300, China; yjyu@zafu.edu.cn

**Keywords:** *Brassica campestris*, CCCH-type zinc-finger protein, BcMF30a, BcMF30c, pollen development, overexpression, cytoplasmic foci

## Abstract

The pollen grains produced by flowering plants are vital for sexual reproduction. Previous studies have shown that two CCCH-type zinc-finger protein genes in *Brassica campestris*, *BcMF30a* and *BcMF30c*, are involved in pollen development. Due to their possible functional redundancy, gain-of-function analysis is helpful to reveal their respective biological functions. Here, we found that the phenotypes of *BcMF30a* and *BcMF30c* overexpression transgenic plants driven by their native promoters were similar, suggesting their functional redundancy. The results showed that the vegetative growth was not affected in both transgenic plants, but male fertility was reduced. Further analysis found that the abortion of transgenic pollen was caused by the degradation of pollen contents from the late uninucleate microspore stage. Subcellular localization analysis demonstrated that BcMF30a and BcMF30c could localize in cytoplasmic foci. Combined with the studies of other CCCH-type genes, we speculated that the overexpression of these genes can induce the continuous assembly of abnormal cytoplasmic foci, thus resulting in defective plant growth and development, which, in this study, led to pollen abortion. Both the overexpression and knockout of *BcMF30a* and *BcMF30c* lead to abnormal pollen development, indicating that the appropriate expression levels of these two genes are critical for the maintenance of normal pollen development.

## 1. Introduction

In flowering plants, each microspore mother cell in the anthers undergoes meiosis to produce four haploid microspores, which are initially associated in a tetrad. Then, the microspores separate from each other and undergo vacuolation and expansion, and the nuclei within them migrate toward the cell wall. Subsequently, pollen mitosis Ⅰ (PMⅠ) yields a bicellular pollen (BCP) composed of a larger vegetative cell (VC) and a smaller generative cell (GC) that is engulfed by VC. The GC further yields two sperm cells after a symmetric division (PMⅡ), and results in a tricellular pollen (TCP) grain. In *Arabidopsis thaliana*, nearly 14,000 genes were identified to be expressed in the male gametophyte, and their expression levels change as development progresses [1]. This clearly illustrates that this series of pollen development events is regulated by dynamic and complex changes in gene expression.

Loss-of-function mutagenesis by fast neutrons, chemicals, or gene editing that create random or targeted mutations or deletions in the genome is a conventional means to uncover the functions of a particular gene [2]. Mutant genetic study has been an effective approach to understand the molecular and cellular aspects of all phases of pollen development. A survey conducted a decade ago showed that 37 genes can affect the post-meiotic male gametophyte development by mutant analysis [3]. After the last ten years of research, gametophytic mutants have been continuously characterized, and genes required for pollen development have been identified exponentially. For instance, *LATERAL ORGAN BOUNDARIES DOMAIN* (*LBD*) *10* co-acts with *SIDECAR POLLEN*/*LBD27* to regulate the early stage of pollen development prior to PMⅠ [4]. An *myb81-1* mutant analysis demonstrated that *MYB81* is a non-redundant GAMYB family member required for polarized microspores’ progress into PMⅠ [5]. Loss of function of *VAC14* led to pollen abortion due to the defective vacuolar fission after PMⅠ [6]. Four genes that encode closely related RING-type E3 ligases, *APD1*–*4*, are redundantly involved in PMⅡ regulation during male gametogenesis [7]. In addition, mutant studies also characterized a large number of genes involved in pollen development by affecting tapetum development and pollen wall formation [8,9].

However, since there are a large number of genes in Arabidopsis, rice, and many other plants that emerge as gene families [10,11], it is not always possible to characterize gene functions only by single-gene mutagenesis. There are many examples showing that mutants with single-gene disruption do not show obvious phenotypes due to genetic redundancy. For instance, Arabidopsis *bHLH010*, *bHLH089*, and *bHLH090* are redundantly required for anther and pollen development meaning that single mutants can be indistinguishable from wild-type plants [12]. What is even more challenging is that it is sometimes difficult to accurately predict how many genes are functionally redundant in the same biological process. As a complementary or alternative approach to the loss-of-function approach, gain-of-function mutagenesis allows the phenotypes of the gain-of-function mutants to be observed without disturbance from other family members, which is conducive to the functional identification of redundant genes [13]. Moreover, the advantages of the gain-of-function approach also include the abilities of (a) identifying genes that confer stress resistance to plants that arise from the introduction of transgenes, e.g., the overexpression transgenic rice plants of a lipid transfer protein, *Oryza sativa* Drought-Induced LTP, were more tolerant to drought stress and showed less tapetal defects during the reproductive stage [14]; and (b) uncovering the functions of genes from non-model plants by using a heterologous expression system, e.g., heterologous overexpression of the stamen-specific R2R3-MYB gene *BcMF28* of *Brassica campestris* in Arabidopsis reveals that it plays a critical regulatory role during late stamen development [15]. In addition, for genes that play a vital regulatory role, the phenotypes of a loss-of-function mutant are often the results of the inactivation of most pathways controlled by the gene, which may conceal the biological process directly regulated by this gene. Therefore, sometimes it may be easier to decipher the exact biological functions of the genes by observing the phenotypes of the gain-of-function mutants [16,17,18]. The *A. thaliana* genome has undergone three paleo-polyploidy events [19]. *Brassica campestris* (syn. *B. rapa*) shares these events but with the addition of a whole-genome triplication (WGT) [20], so most genes contain more duplications, which makes it more difficult to characterize gene functions only through the loss-of-function approach.

Cysteine3Histidine (CCCH)-type zinc-finger proteins form a large family that is conserved across eukaryotes. In plants, the number of tandem CCCH-type zinc-finger (TZF) proteins containing two zinc-finger motifs is usually the largest. In Arabidopsis, the TZF proteins with an arginine-rich (RR) region ahead of the TZF motif constitute the largest subfamily (RR-TZF), with a total of 11 members (AtTZF1–11) [21]. Up to now, hundreds of genes have been determined to function in various developmental events during male gametogenesis, either by loss-of-function or gain-of-function approaches. We previously identified eleven male fertility-related CCCH-type zinc-finger protein genes in Chinese cabbage, including a pair of paralogs expressed in pollen during male gametophyte development, named *Brassica campestris Male Fertility 30a* (*BcMF30a*) and *BcMF30c* [22]. BcMF30a and BcMF30c are two non-TZF proteins that contain a CCCH motif and two putative RNA-binding domains (RBDs), RRM and LOTUS. Genetic analysis of the double mutants constructed using CRISPR-Cas9 gene editing technology showed that partial pollen were aborted due to the degradation of pollen inclusions during microgametogenesis. However, the impact of each gene on pollen development and whether there is functional differentiation between *BcMF30a* and *BcMF30c* still remains to be ascertained.

Here, we analyzed the expression of *BcMF30a* and *BcMF30c* in seedlings and various tissues during reproductive growth. We also used one of the gain-of-function approaches, i.e., overexpressing coding sequences of *BcMF30a* and *BcMF30c* by their native promoters, to further investigate their biological functions during male gametogenesis. The results showed that overexpression of *BcMF30a* or *BcMF30c* could affect pollen development after microspore formation, and eventually led to pollen abortion. We also speculated that the phenotypes caused by the overexpression may be related to the variable subcellular localization properties of BcMF30a and BcMF30c.

## 2. Materials and Methods

### 2.1. Plant Material and Growth Conditions

*Brassica campestris* L. ssp. *chinensis* var. *parachinensis* cv. Youqing 49 was used for the genetic transformation. Chinese cabbage and Arabidopsis were planted in a phytotron at 20 ± 2 °C under a 16/8-h light/dark cycle. Seven-day-old Arabidopsis seedlings (for GUS histochemical staining assay) were grown on synthetic Murashige and Skoog (MS) medium containing 2% Suc and 0.8% agar in a 22 °C growth chamber under a 14-h-light/10-h-dark regime. The tobacco (*Nicotiana benthamiana*) plants were grown in the growth room at 26 ± 2 °C under a 16/8-h light/dark cycle. 

### 2.2. β-glucuronidase (GUS) Histochemical Staining Assay

The *ProBcMF30a:GUS* and *ProBcMF30c:GUS* transgenic Arabidopsis plants were generated earlier [22]. GUS staining of floral buds and other tissues was performed as described by Mudunkothge et al. [23]. Anther stages of Arabidopsis were referred to as described [24]. Images were obtained using a Nikon microscope (ECLIPSE 90i, Nikon, Tokyo, Japan).

### 2.3. Generation of BcMF30a and BcMF30c Overexpression Transgenic Chinese Cabbage

To construct the *BcMF30a* and *BcMF30c* overexpression vectors, the coding regions of *BcMF30a* (1608-bp) and *BcMF30c* (1602-bp) were amplified and subcloned into the reconstructed binary vectors, *ProBcMF30a:GUS* and *ProBcMF30c:GUS*, which have been constructed in our previous study [22]. Then, the recombinant vectors *ProBcMF30a:BcMF30a* and *ProBcMF30c:BcMF30c* were introduced into Chinese cabbage by the *Agrobacterium*-mediated transformation method as described previously [25]. The positive transformants were identified by the extraction of their DNA and analyzed by PCR screening and sequencing. For partial transgenic T_0_ line plants, the positive T_1_ plants were propagated and identified. The primers used are listed in Table S1.

### 2.4. RNA Extraction and qRT-PCR

Total RNA was extracted from inflorescences of transgenic Chinese cabbage by using RNAiso Plus (Takara, Kyoto, Japan). A PrimerScript RT reagent Kit (Takara, Kyoto, Japan) was used for preparing cDNA. A SYBR^®^ Premix Ex Taq™ Kit (Takara, Kyoto, Japan) was used for quantitative PCR, which was conducted on a CFX96 Real-Time System (Bio-Rad, Hercules, CA, USA). The gene encoding protein of ubiquitin conjugating enzyme (UBC10) was selected as a reference gene to normalize the quantity of total RNA. The qRT-PCR was carried in three technical replicates, and the relative gene expression levels were calculated using the 2^−ΔΔCt^ method. All primers used are listed in Table S1.

### 2.5. Phenotypic Analyses, Cytological Observation, and Pollen Germination Assay

Pollen viability was investigated by staining with Alexander solution [26]. In vitro and in vivo pollen germination tests, scanning electron microscopy (SEM), and transmission electron microscopy (TEM) analyses were conducted as described by Lin et al. [27] and Xu et al. [22], with minor modifications. The images of Alexander-stained pollen grains, germinated pollens, and fertilized pistils were captured by using a fluorescent microscope (ECLIPSE 90i, Nikon, Japan).

### 2.6. Subcellular Localization

In our previous studies, we constructed vectors for the subcellular localization experiments, namely *pFGC-eGFP-BcMF30a* and *pFGC-eGFP-BcMF30c* [22]. The fusion vectors were transiently transformed into leaf epidermal cells of H2B-RFP transgenic tobacco plants by using an infiltrated method. The subcellular localization of eGFP-fusion proteins was analyzed 48 h after introduction under a confocal laser scanning microscope (A1, Nikon, Tokyo, Japan) with the NIS-elements AR software (Nikon) using ND acquisition with Z movement before and after heat treatment (42 °C for 90 min).

## 3. Results

### 3.1. BcMF30a and BcMF30c Share Similar Gene Expression Patterns and Both Are Highly Expressed in Bicellular Pollen

**Figure 1:**
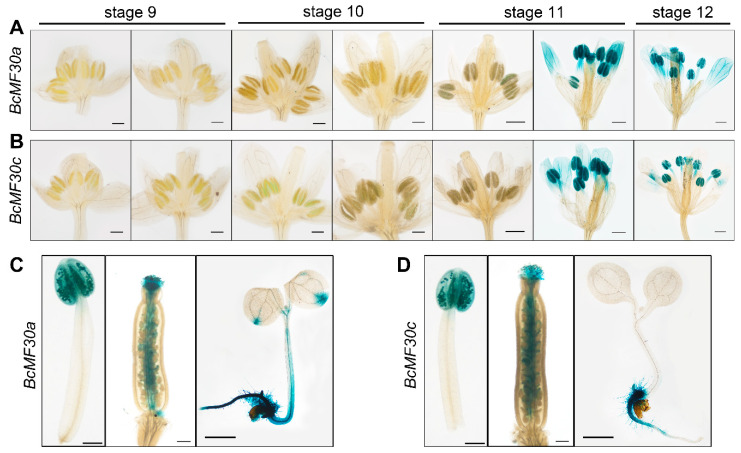
Spatiotemporal expression patterns of *BcMF30a* and *BcMF30c*. β-glucuronidase (GUS) staining of floral buds of *ProBcMF30a:GUS* (**A**) and *ProBcMF30c:GUS* (**B**) plants at different stages of pollen development. GUS staining of anthers, fertilized pistils, and 7-day-old seedlings of *ProBcMF30a:GUS* (**C**) and *ProBcMF30c:GUS* (**D**) plants.

Previous studies in our lab have shown that both *BcMF30a* and *BcMF30c* are mainly expressed in pollen and pollen tubes [22]. In order to further determine at which stage of pollen development they are expressed, and whether they are expressed in other tissues, we used previously obtained transgenic plants containing *ProBcMF30a:GUS* or *ProBcMF30c:GUS* for further expression analysis. As shown in Figure 1A,B, the GUS signals in anthers reached their peak at late stage 11 and stage 12, indicating that the promoter activity of *BcMF30a* and *BcMF30c* promoters reached the highest point in anthers at the late binucleate and trinucleate stages. Since GUS histochemical staining often contaminates surrounding tissues, we performed GUS staining on each flower organ separately. For both *BcMF30a* and *BcMF30c*, GUS activity could be detected only in anthers and fertilized pistils (Figure 1C,D), but there was no GUS signal in sepals, petals, and unfertilized pistils (Figure S1). Roots of 7-day-old seedlings also showed clear GUS staining. Nevertheless, the promoter activity could be detected on the apex of cotyledons of *ProBcMF30a:GUS* seedlings, but not on that of *ProBcMF30c:GUS* seedlings (Figure 1C,D). Additionally, no GUS staining was observed in leaves, stems, and siliques (Figure S1).

### 3.2. Overexpression of BcMF30a and BcMF30c in B. campestris Causes Reduced Male Fertility

**Figure 2:**
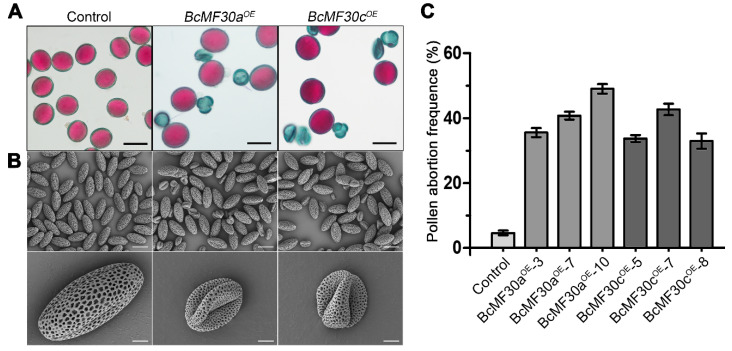
Cytological observation of mature pollen grains from *BcMF30a^OE^ and BcMF30c^OE^* transgenic plants of *Brassica campestris*. Alexander staining (**A**) and scanning electron micrograph (SEM) observation (**B**) of *BcMF30a^OE^ and BcMF30c^OE^* mature pollen grains. (**C**) Analysis of pollen abortion frequency in the T_1_ lines of *BcMF30a^OE^ and BcMF30c^OE^* plants. The values are the mean ± SD (standard deviation). Bars = 25 μm in (**A**), and 30 and 5 μm in (**B**).

In the previous study, we constructed double-knockout mutants of *BcMF30a* and *BcMF30c* in Chinese cabbage and showed that the lack of expression of these two genes could lead to partial pollen abortion and abnormal pollen germination [22]. To further explore the biological functions of these two genes during pollen development, transgenic plants with *BcMF30a* and *BcMF30c* coding sequences driven by their native promoters were used for overexpression analysis. A total of 12 and 8 positive T_0_ transgenic lines (named *BcMF30a^OE^* and *BcMF30c^OE^*, respectively) were generated after introducing the constructs of *ProBcMF30a:BcMF30a* and *ProBcMF30c:BcMF30c* into Chinese cabbage calli. Since *BcMF30a* and *BcMF30c* were mainly expressed in pollen, we first paid attention to whether the pollen fertility of these transgenic plants was affected. Alexander staining revealed a reduction (14.1–46.2%) in the viability of pollen grains produced by 11 of 12 *BcMF30a^OE^* (except *BcMF30a^OE^-9*) and all *BcMF30c^OE^* T_0_ lines (Figure S2A). Of these, *BcMF30a^OE^-1*, *3*, *7*, *8*, and *10* and *BcMF30c^OE^-1*, *5*, *7*, and *8* showed the most significant reduction (over 30%) of pollen viability when compared with the control plants. Thus, the progenies (T_1_ plants) of *BcMF30a^OE^-3*, *7*, and *10*, and *BcMF30c^OE^-5*, *7*, and *8* were chosen for further analysis. As expected, all T_1_ plants still showed that more than 30% of pollen grains were nonviable (Figure 2A,C). SEM analysis also revealed that these aborted pollen grains shriveled and collapsed severely (Figure 2B).

The *BcMF30a* and *BcMF30c* mRNA expression levels in *BcMF30a^OE^* and *BcMF30c^OE^* plants were measured. Interestingly, qRT-PCR analysis revealed that no significant increase in mRNA expression was detected, and there was even a decrease in the mRNA level of *BcMF30c* in *BcMF30c^OE^-7* (Figure S2B,C). This may be due to the abortion of pollen grains, and the specificity and relatively low activation efficiency of native promoters. Since *BcMF30a* and *BcMF30c* are only expressed in developing pollen and pollen tubes during the reproductive growth stage, it is reasonable and predictable that these transgenic plants had normal vegetative growth and floral morphology (Figure S3).

### 3.3. The Degradation of Microspore Contents Starts from the Late Uninucleate Stage in BcMF30a^OE^ and BcMF30c^OE^ Transgenic Plants

**Figure 3:**
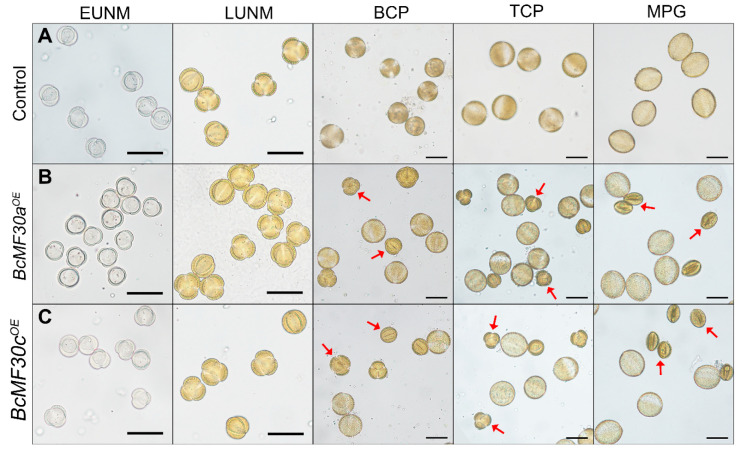
The pollen grains produced by *BcMF30a^OE^* and *BcMF30c^OE^* transgenic plants at different developmental stages were observed by optical electron microscope. Pollen grains produced by the control plants (**A**), *BcMF30a^OE^* plants (**B**), and *BcMF30c^OE^* plants (**C**). Red arrows indicate the pollens with abnormal development. EUNM, early uninucleate microspore; LUNM, late uninucleate microspore; BCP, bicellular pollen; TCP, tricellular pollen; MPG, mature pollen grain. Bars = 20 μm.

Light microscopy was used to evaluate the morphological changes of microspores during pollen development. No difference was observed between the microspores produced by the control and the transgenic plants during the microsporogenesis phase. However, at the stage of microgametogenesis, some bicellular pollen produced by the transgenic plants were obviously morphologically abnormal, and then further shriveled and aborted (Figure 3).

**Figure 4:**
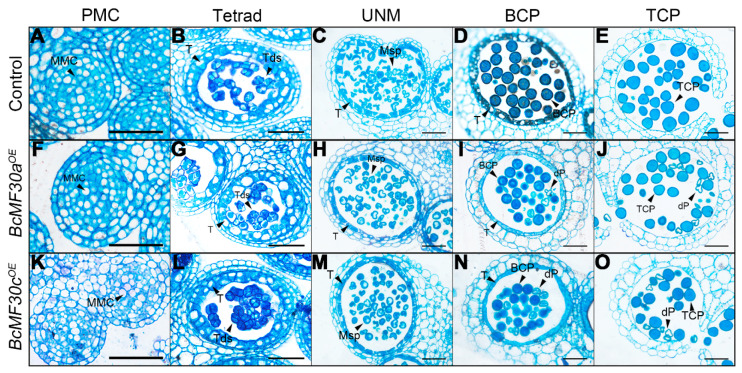
Semi-thin transverse sections of anthers from *BcMF30a^OE^ and BcMF30c^OE^* plants of *Brassica campestris*. Semi-thin sections of anthers from the control (**A**–**E**), *BcMF30a^OE^* (**F**–**J**), and *BcMF30c^OE^* (**K**–**O**) plants at the pollen mother cell stage (PMC, **A**,**F**,**K**), tetrad stage (Tetrad, **B**,**G**,**L**), uninucleate microspore stage (UNM, **C**,**H**,**M**), bicellular pollen stage (BCP, **D**,**I**,**N**), and tricellular pollen stage (TCP, **E**,**J**,**O**). BCP, bicellular pollen; dP, degenerated pollen; MMC, microspore mother cell; Msp, microspore; T, tapetum; TCP, tricellular pollen; Tds, tetrads. Bars = 25 μm.

A semi-thin transverse section was conducted to investigate the precise stage at which pollen abnormality begins. No obvious defects were detected inside both *BcMF30a^OE^* and *BcMF30c^OE^* anther locules from the pollen mother cell stage to the uninucleate stage. However, during the binucleate stage, visible abnormalities appeared in these anthers, in which parts of pollen were deformed. Subsequently, the pollen contents continued to degrade until they disappeared (Figure 4).

**Figure 5:**
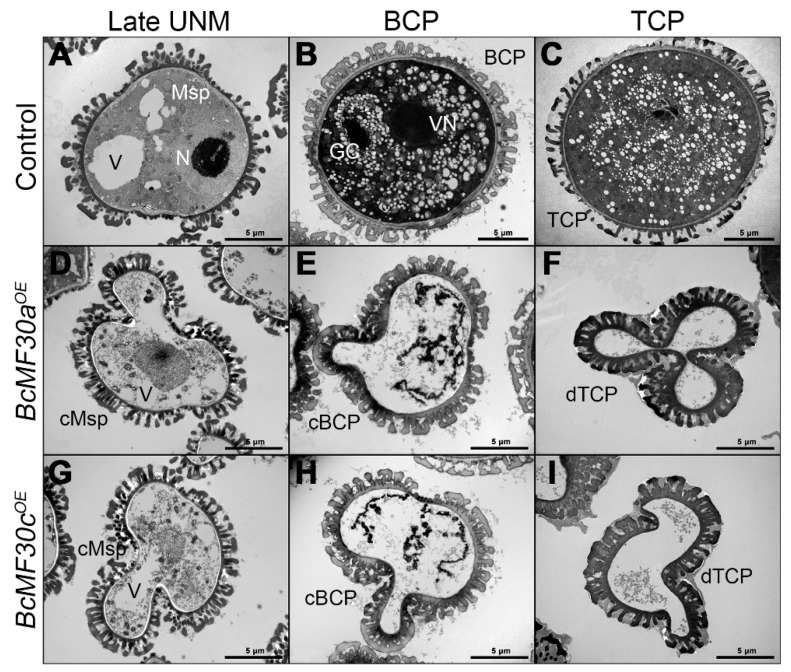
Transmission electron microscopy (TEM) observation of pollen grains from *BcMF30a^OE^* and *BcMF30c^OE^* plants of *Brassica campestris*. Ultrastructure of pollen grains at different developmental stages from the control (**A**–**C**), *BcMF30a^OE^* (**D**–**F**), and *BcMF30c^OE^* (**G**–**I**) plants. (**A**,**D**,**G**), late uninucleate stage; (**B**,**E**,**H**), bicellular stage; (**C**,**F**,**I**), tricellular stage. BCP, bicellular pollen; cBCP, collapsed BCP; GC, generative cell; Msp, microspore; cMsp, collapsed Msp; N, nucleus; TCP, tricellular pollen; dTCP, degenerated TCP; V, Vacule; VN, vegetative nucleus. Bars = 5 μm.

TEM was further performed to confirm the degradation process. As shown in Figure 5, the differences between the control pollen and *BcMF30a^OE^* or *BcMF30c^OE^* pollen appeared from the late uninucleate stage onwards. By this stage, the round nucleus in the control microspore was moved to the side by the large vacuole. However, the *BcMF30a^OE^* and *BcMF30c^OE^* microspores were concave in shape, with reduced cytoplasmic contents and no nuclei. At the binucleate stage, the control microspore underwent an asymmetrical mitosis and became a bicellular pollen with a generative cell located in the cytoplasm of the vegetative cell. By contrast, the cellular structures and contents of transgenic pollen further degraded. By the trinucleate stage, the mature pollen grains produced by the control plants were well developed with a dense cytoplasm, whereas the *BcMF30a^OE^* and *BcMF30c^OE^* pollen shrank severely due to the degradation of the cytoplasm, leaving only the pollen wall that seemed to be intact.

### 3.4. In Vitro and In Vivo Pollen Germination Tests in BcMF30a^OE^ and BcMF30c^OE^ Transgenic Plants

**Figure 6:**
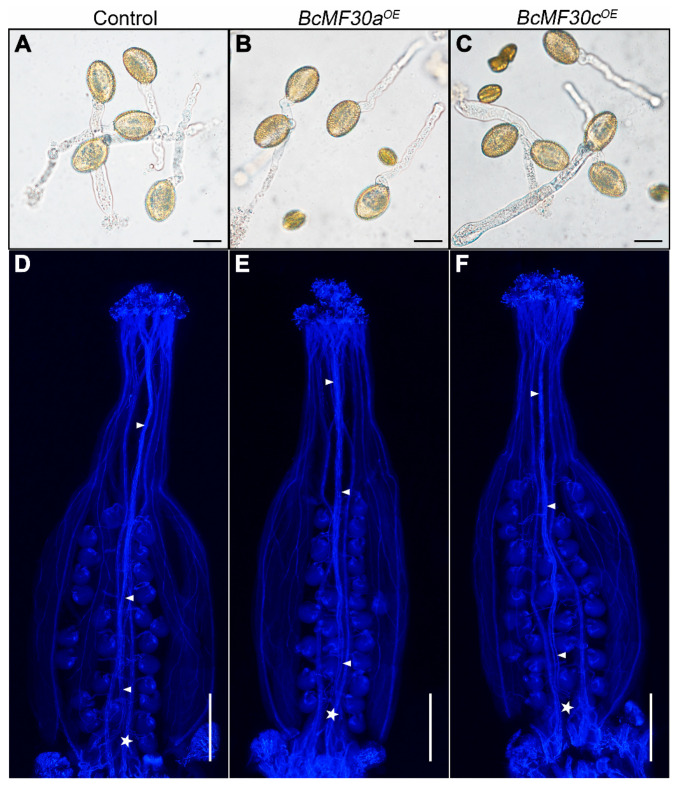
The in vitro and in vivo pollen germination tests of *BcMF30a^OE^* and *BcMF30c^OE^* transgenic plants. (**A**–**C**) The in vitro pollen germination test. (**D**–**F**) The in vivo pollen germination and pollen tube growth. The arrows indicate the pollen tubes, and the asterisks indicate the end position of pollen tubes. Bars = 20 μm in (**A**–**C**); 100 μm in (**D**–**F**).

In vitro and in vivo pollen germination experiments were conducted to investigate the differences in pollen germination performance. In vitro, all shrunken pollen grains in both *BcMF30a^OE^* and *BcMF30c^OE^* plants failed to germinate as expected (Figure 6A–C). Therefore, only the plump spindle-shaped pollen grains in transgenic plants were counted for germination rate statistics. The frequencies of the pollen germination of the *BcMF30a^OE^* (89.3%) and *BcMF30c^OE^* plants (87.6%) were near to those of the control plants (91.2%). There was also no significant difference in pollen tube growth between the transgenic plants and the control plants in vivo. The results revealed that the numbers of pollen tubes that grew in *BcMF30a^OE^* and *BcMF30c^OE^* pistils were equivalent to those in the control plants after self-pollination (Figure 6D–F). These observations indicated that although the overexpression of *BcMF30a* and *BcMF30c* caused partial pollen to fail to germinate due to abortion, it did not affect the pollination and fertilization process of other viable pollen grains.

### 3.5. BcMF30a and BcMF30c Can Localize to Cytoplasmic Foci

BcMF30a and BcMF30c were reported to be located in both the nucleus and the cytoplasm under normal conditions in tobacco epidermal cells in a previous study [22]. Several studies have revealed that all AtTZFs and multiple other CCCH-type zinc-finger proteins can be localized in cytoplasmic foci similar to processing bodies (PBs) and stress granules (SGs) [28,29,30]. In order to investigate whether BcMF30a and BcMF30c were related to the cytoplasmic foci like PBs and SGs under certain conditions, the tobacco transient expression system was once again used to analyze their subcellular localization.

**Figure 7:**
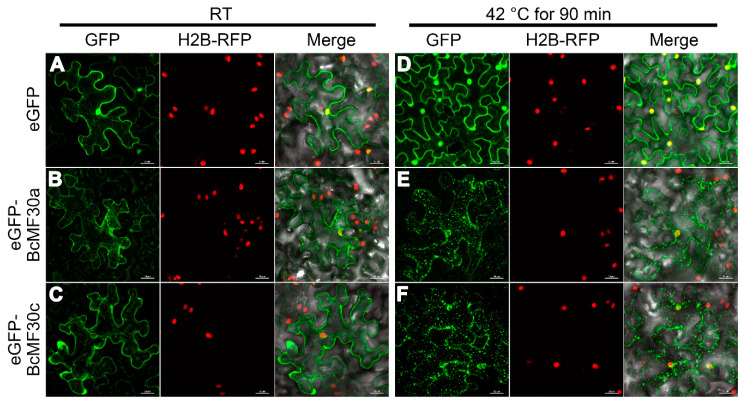
BcMF30a and BcMF30c can form cytoplasmic foci in *Nicotiana benthamiana* leaf epidermal cells after heat treatment. eGFP, eGFP-BcMF30a, and eGFP-BcMF30c were dispersed in the cytoplasm at room temperature (RT) (**A**–**C**). After heat treatment at 42 °C for 90 min, eGFP-BcMF30a and eGFP-BcMF30c could form cytoplasmic foci (**E**,**F**), while no cytoplasmic foci were observed for eGFP (**D**). H2B is a marker protein of the nucleus. Pictures represent white field images (Bright), epifluorescence (GFP and RFP), and merged images (Merge). Bar =25 μm.

Since heat stress can usually promote or induce the formation of cytoplasmic foci like PB and SG [31], we observed and compared the localization patterns of the transiently expressed GFP fusion proteins in the epidermal cells of *H2B-RFP* (nuclear localization protein) transgenic tobacco before and after heat treatment. Excitingly, the heat stress changed both eGFP-BcMF30a and eGFP-BcMF30c from being mainly dispersed in the cytoplasm to being aggregated into cytoplasmic foci (Figure 7E,F). In contrast, the free-eGFP still remained as diffused localization after heat stress (Figure 7D). These results indicated that BcMF30a and BcMF30c exhibited variable subcellular localization characteristics and may play roles in mRNA regulation like PBs and SGs.

## 4. Discussion

The plant CCCH-type zinc-finger proteins play important regulator roles in multiple biological processes, such as the regulation of plant growth and development [32,33,34], adaptation to stress and hormone responses [35,36,37,38,39,40,41,42,43,44,45], and acquisition of immunity against pathogens [46,47,48]. It is worth noting that the biological functions of many CCCH-type zinc-finger protein genes were confirmed by constructing and observing the phenotypes of overexpression transgenic plants, since many single loss-of-function mutants of CCCH-type zinc-finger genes showed a subtle or no phenotype. Interestingly, although the overexpression of many CCCH-type zinc-finger genes is helpful for cells to resist certain stresses, there are some transgenic plants that show growth defects [30,36,40,41,49]. For example, constitutive overexpression of *AtC3H14* and *AtC3H15/CDM1* in Arabidopsis resulted in a dwarf phenotype and male sterility, respectively [33,50]. In this study, we also found that overexpressing *BcMF30a* or *BcMF30c* in Chinese cabbage plants can lead to abnormal pollen development. In order to avoid the impact of ectopic expression on vegetative growth, we used their native promoters to drive overexpression instead of the constitutive promoter *CaMV35S*, which is commonly used in overexpression experiments. This was also because we used native promoters to ensure that *BcMF30a* and *BcMF30c* were not expressed in tissues other than pollen in the floral buds, meaning that we have not detected a significant increase in gene expressions in the inflorescence of transgenic plants. Moreover, some plants even showed a decline in gene expressions due to a large amount of pollen abortion (Figure S2B,C). We observed that the pollen development was disrupted both in *BcMF30a^OE^* and *BcMF30c^OE^* transgenic plants. Combined with the optical electron microscope observation, semi-thin section, and ultra-thin section results, it was found that the overexpression of *BcMF30a* and *BcMF30c* led to the abnormal pollen development from late uninucleate microspores (Figure 3, Figure 4 and Figure 5). Intriguingly, the pollen abortion phenotype caused by the overexpression of these two genes is very similar to the phenotype of their double mutants [22], indicating that the appropriate expression levels of these two genes are essential for maintaining normal male gametogenesis. In addition, since there was no significant difference between the phenotypes of *BcMF30a^OE^* and *BcMF30c^OE^* plants, we believed that there was a great functional redundancy between these two genes during pollen development. However, there were small differences in the expression patterns in seedlings, in which *BcMF30a* could be expressed at the cotyledon apex, while *BcMF30c* could not (Figure 1C,D), indicating that they might have undergone functional differentiation in the morphological construction of the cotyledon apex.

Many studies have shown that the overexpression plants of multiple CCCH-type zinc-finger protein genes (e.g., *AtTZF1*–*6*, *AtC3H14,* and *AtC3H15*/*CDM1*, and *BcMF30a and BcMF30c* in this study) exhibit abnormal growth and development. Does this indicate that there is a general molecular mechanism behind the functions of these genes? Excitingly, multiple studies have found that many TZF proteins displayed special and similar subcellular localization patterns. It has been reported that all members of RR-TZFs (AtTZF1–11) and another two TZFs (AtC3H14 and AtC3H15/CDM1) in Arabidopsis could localize to cytoplasmic foci in maize protoplasts [29]. Arabidopsis AtTZF1, 4, 5, 6, and 9, and rice OsTZF1 have also been shown to be co-localized with the markers of PBs and SGs [30,47,51]. PBs and SGs are two distinct but closely related mRNP granules which are enriched with RNA-binding proteins and translation-repressed or translationally stalled mRNAs, and are important for RNA regulation [31,52,53]. Moreover, it has been found that overexpression of TZFs, some stresses (e.g., salt stress), and hormone treatment (e.g., jasmonic acid and abscisic acid) can induce the formation of TZF-associated cytoplasmic foci [30,39,51]. In this study, we did find that the heat treatment could induce the formation of BcMF30a/BcMF30c-associated cytoplasmic foci in tobacco epidermal cells (Figure 7), indicating that the properties of these two CCCH-type zinc-finger proteins may be similar to AtC3H14, AtC3H15/CDM1, and AtTZFs in Arabidopsis. This also implies that overexpression of BcMF30a and BcMF30c may induce the formation of cytoplasmic foci. This hypothesis was also supported by the research of AtC3H18L, a novel CCCH-type zinc-finger protein identified recently [28]. Studies have shown that AtC3H18L could not only form heat stress-induced cytoplasmic foci in tobacco epidermal cells and co-localize with PB and SG markers, but it could also form cytoplasmic foci in overexpressed transgenic Arabidopsis under normal growth conditions. In mammalians, the overexpression of PB components can induce the formation of abnormally large PBs, which behave abnormally and exert aberrant function [54]. Therefore, we speculated that it might be the overexpression of BcMF30a and BcMF30c in microspores that led to the continuous formation of abnormal cytoplasmic foci, which disrupted the balance of mRNAs and ultimately resulted in pollen abortion. We believed that this hypothesis may also be one of the explanations for the growth inhibition and developmental defects of transgenic plants overexpressing TZFs. However, more evidence is needed to support this hypothesis. In addition, is the enhanced stress resistance of many overexpression transgenic plants of CCCH-type zinc-finger proteins related to the cytoplasmic foci formed by these proteins? Can overexpression of BcMF30a and BcMF30c also enhance stress resistance in a certain aspect? These issues are urgent and worthy of being revealed. 

It has been proved that the TZF motif with RNA binding function in AtTZF9, rather than the putative protein–protein interaction ankyrin repeat motif, was partially responsible for its cytoplasmic foci localization [47]. Although there is just one CCCH-type motif in BcMF30a, BcMF30c, and AtC3H18L, they all contain two putative RBDs—RRM and LOTUS [22,28]—which indicates that these two RBDs may be responsible for their specific subcellular localization patterns. Further research is needed to clarify this.

## 5. Conclusions

The gain-of-function approach is one of the excellent methods for studying genes with redundant functions. Here, we determined that the specific overexpression of BcMF30a or BcMF30c could result in pollen abortion due to the degradation of pollen contents from the late uninucleate microspore stage. Further analysis revealed that these two proteins could form cytoplasmic foci like TZFs in Arabidopsis. Combined with the existing research of TZFs, we speculated that the high overexpression of these two proteins could induce the persistent formation of cytoplasmic foci, which would lead to pollen abortion. Together with the results that double mutants also exhibited male sterility, this suggests that appropriate expression levels of *BcMF30a* and *BcMF30c* are critical for maintaining normal pollen development. Future work should explore the functions of *BcMF30a* and *BcMF30c* in greater depth to unravel the mechanisms regulating male gametophyte development in Chinese cabbage.

## Supplementary Materials

The following are available online at https://www.mdpi.com/2073-4425/11/11/1287/s1, Figure S1: *BcMF30a* and *BcMF30c* were not expressed in some floral organs and tissues, Figure S2: Analysis of pollen abortion frequency and gene expression levels in *BcMF30a^OE^* and *BcMF30c^OE^* transgenic plants of *Brassica campestris*, Figure S3: Morphological observation of plants and floral organs of *BcMF30a^OE^ and BcMF30c^OE^* transgenic plants of *Brassica campestris*, Table S1: Sequences of primers used in this study.
