# Supplementary material for: Overexpression of Two CCCH-type Zinc-Finger Protein Genes Leads to Pollen Abortion in Brassica campestris ssp. chinensis
Source: Genes (Basel). 2020 Oct 29;11(11):1287. doi: 10.3390/genes11111287 (PMC7693475; doi:10.3390/genes11111287)
Supplement: Supplementary file 1 [file genes-11-01287-s001.pdf]

genes

MDPI

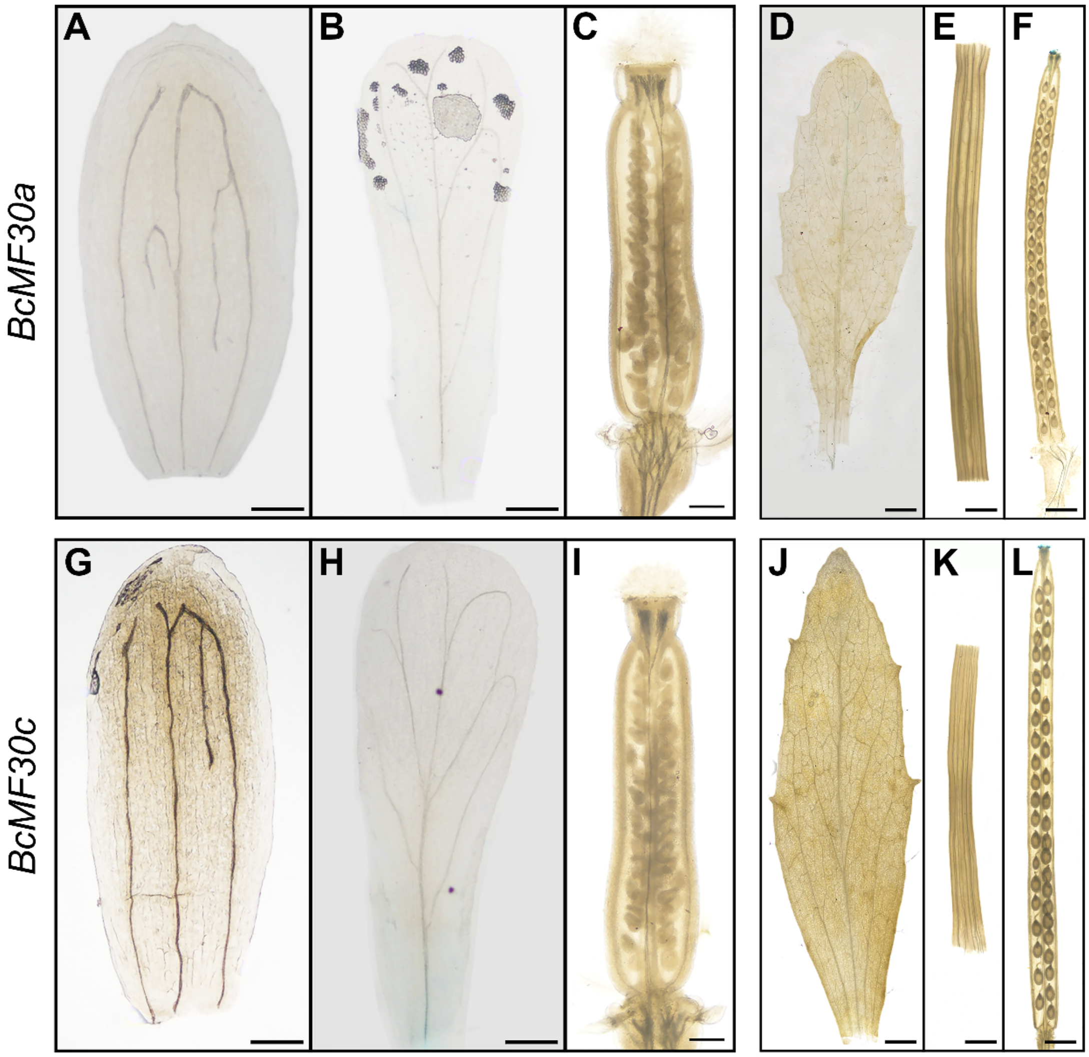

Figure S1 *BcMF30a* and *BcMF30c* were not expressed in some floral organs and tissues. No GUS signal was observed in the sepal (**A,G**), petal (**B,H**), unfertilized pistil (**C,I**), leaf (**D,J**), stem (**E,K**) and silique (**F,L**) of *ProBcMF30a:GUS* (**A**—**F**) and *ProBcMF30c:GUS* (**G**—**L**) plants. Bar = 200 μm in (**A**—**C**,**G**—**I**), 1 mm in (**D**—**F**,**J**—**L**).

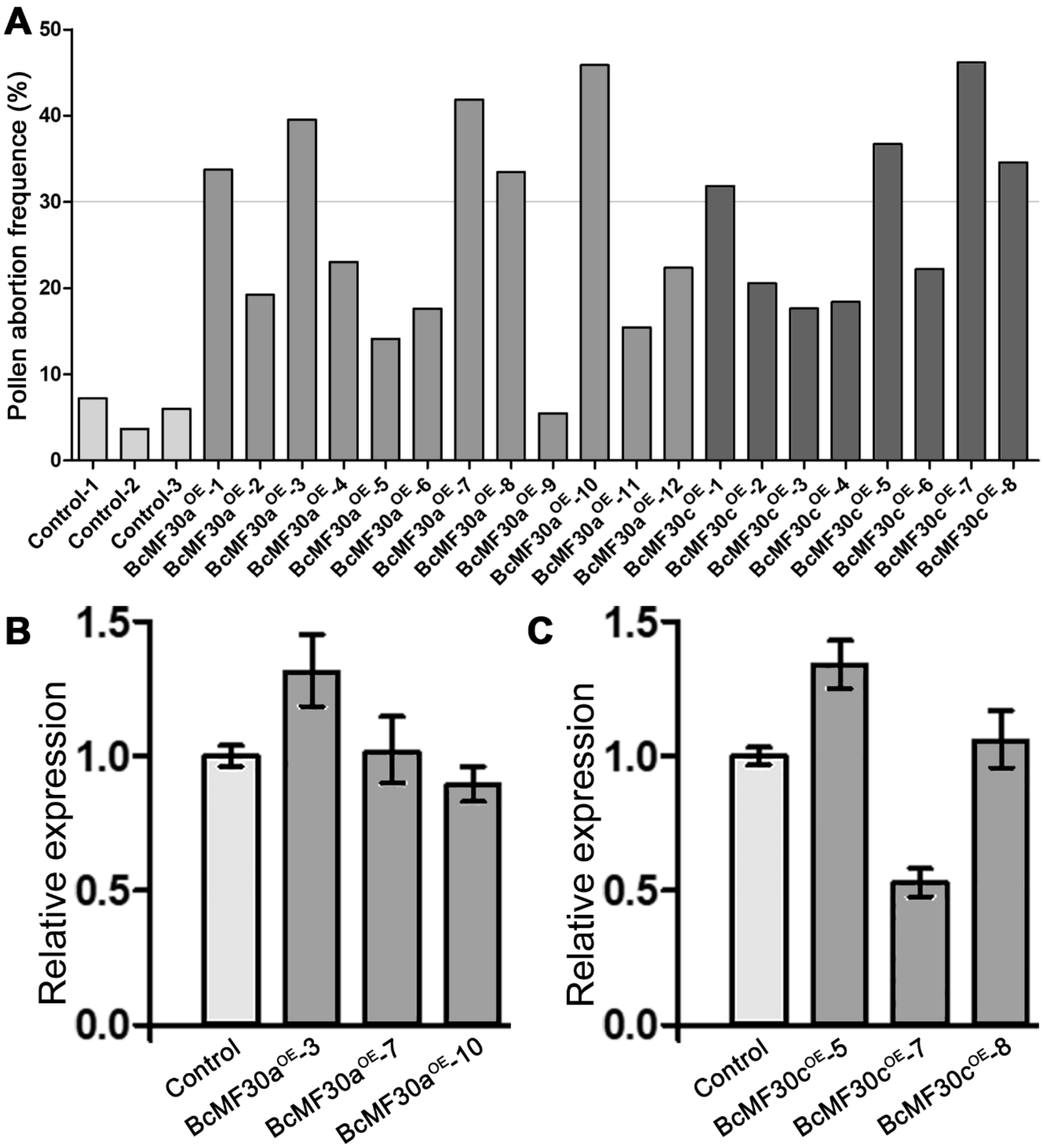

Figure S2 Analysis of pollen abortion frequency and gene expression levels in *BcMF30a*^{OE} and *BcMF30c*^{OE} transgenic plants of *Brassica campestris*. (**A**) The pollen abortion frequency analysis of 12 and 8 positive transgenic T_0 lines of *BcMF30a*^{OE} and *BcMF30c*^{OE} transgenic plants. qRT-PCR showed the expression of *BcMF30a* (**B**) and *BcMF30c* (**C**) in three T_1 lines of *BcMF30a*^{OE} and *BcMF30c*^{OE} transgenic plants, respectively. Ubiquitously expressed *UBC10* was used as reference gene.

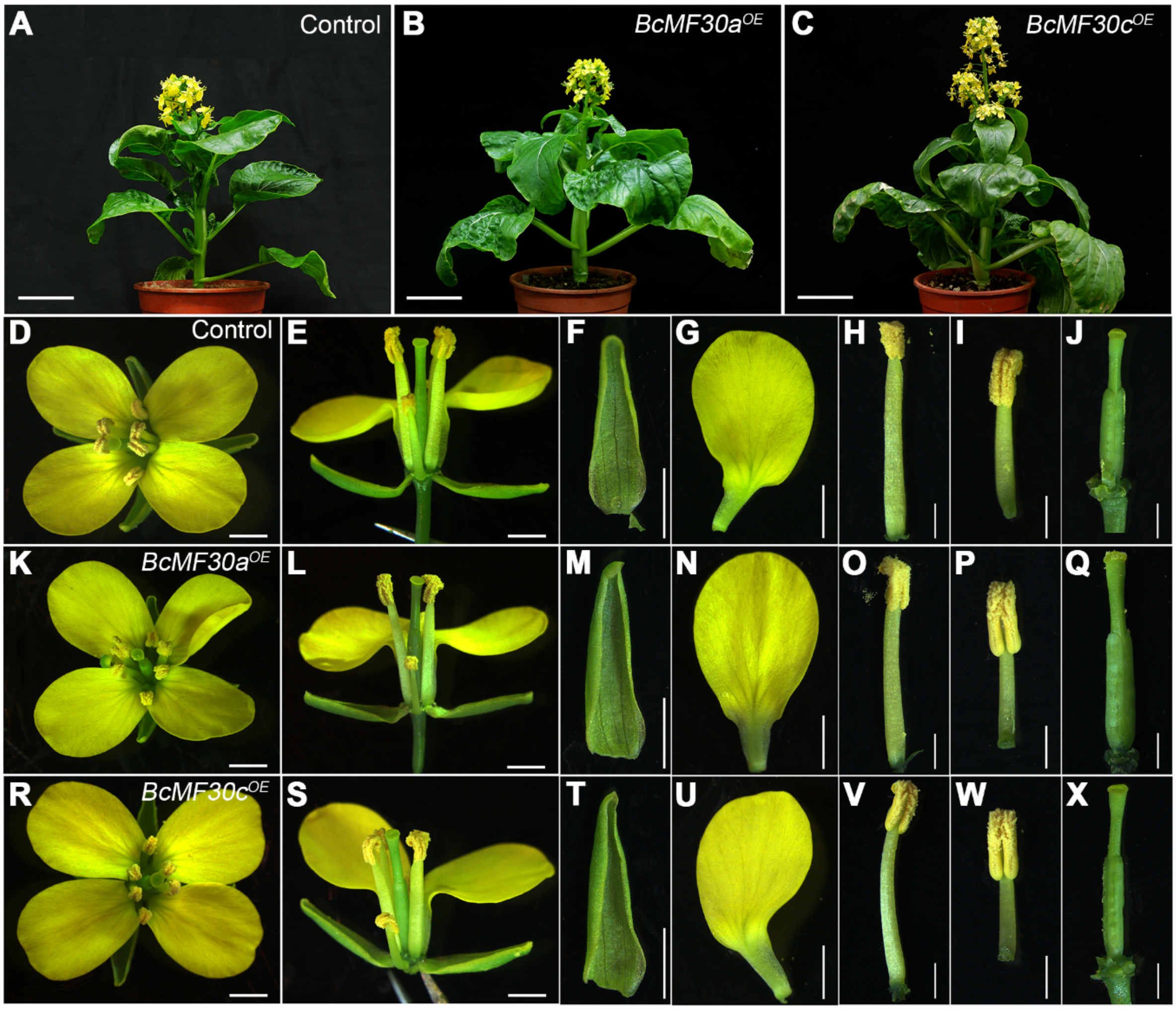

Figure S3 Morphological observation of plants and floral organs of $BcMF30a^{OE}$ *and* $BcMF30c^{OE}$ transgenic plants of *Brassica campestris*. (**A**), (**B**) and (**C**) showed the plants of control, $BcMF30a^{OE}$ and $BcMF30c^{OE}$, respectively. (**D**—**J**), (**K**—**Q**) and (**R**—**X**) indicated the flower, sepal, petal, stamens and pistil of the control, $BcMF30a^{OE}$ and $BcMF30c^{OE}$, respectively. Bars = 5 cm in (**A**—**C**), 2 mm in (**D**—**X**).

Table S1 Sequences of primers used in this study.

Experiment	Primer Name	Sequence-F(5'-3')	Sequence-R(5'-3')
BcMF30a CDS	BcMF30a-1F/R	ATTATTGGAGTCTAGAATGAATTT CACAGAATCGAT	GACCACCCGGGGATCCCTAGGTAA CTGTCGAAATCTC
BcMF30c CDS	BcMF30c-1F/R	ATTATTAGAGTCTAGAATGAATTT CACAGAATCTATGAAC	GACCACCCGGGGATCCCTATGTAA CTGTTGAAATCTCCTT
BcMF30a^{OE} Test	BcMF30a-2F/R	AGGACGGTTGTGCTATCT	GGCTTCAAATGGCGTAT
BcMF30c^{OE} Test	BcMF30c-2F/R	AACGCAATTAATGTGAGTTAGC	GGCAAACAAAACTTGGAGG
BcMF30a qRT-PCR	BcMF30a-3F/R	ATTGCCACCACGAGTTAGC	TCCTGAAGTCATCGGAGAA
BcMF30c qRT-PCR	BcMF30c-3F/R	TGCCAGCGCGTGTAAGT	CCGCATCTGCTGTTAGTTTA
UBC10 qRT-PCR	UBC10-F/R	GGGTCCTACAGACAGTCCTTAC	ATGGAACACCTTCGTCCTAAA
